# Filoviruses: One of These Things is (not) Like the Other

**DOI:** 10.3390/v7102867

**Published:** 2015-09-29

**Authors:** Scott M. Anthony, Steven B. Bradfute

**Affiliations:** 1 Immunology Graduate Program, The University of Texas Graduate School of Biomedical Sciences at Houston, Department of Immunology, The University of Texas MD Anderson Cancer Center, Houston, TX 77030, USA; E-Mail: scottmanthony@gmail.com; 2 University of New Mexico, Center for Global Health, Department of Internal Medicine, MSC10 5550, 1 University of New Mexico, Albuquerque, NM 87131, USA

**Keywords:** Ebola, Ebolavirus, Marburg, Marburgvirus, *Filoviridae*, filovirus, hemorrhagic, animal models, correlates of immunity

## Abstract

The family *Filoviridae* contains several of the most deadly pathogens known to date and the current Ebola virus disease (EVD) outbreak in Western Africa, due to Ebola virus (EBOV) infection, highlights the need for active and broad research into filovirus pathogenesis. However, in comparison, the seven other known filovirus family members are significantly understudied. Many of these, including Marburgviruses and Ebolaviruses other than EBOV, are also highly virulent and fully capable of causing widespread epidemics. This review places the focus on these non-EBOV filoviruses, including known immunological and pathological data. The available animal models, research tools and currently available therapeutics will also be discussed along with an emphasis in the large number of current gaps in knowledge of these less highlighted filoviruses. It is evident that much research is yet to be done in order to bring the non-EBOV filovirus field to the forefront of current research and, importantly, to the development of more effective vaccines and therapeutics to combat potential future outbreaks.

## 1. Introduction

Filoviruses are among the most lethal human pathogens in the world. There are three filovirus genera: *Ebolavirus* (members: Ebola virus (EBOV), Sudan virus (SUDV), Reston virus (RESTV), Taï Forest virus (TAFV), and Bundibugyo virus (BDBV)); *Marburgvirus* (Marburg virus (MARV) and Ravn virus (RAVV)); and *Cuevavirus* (Lloviu virus (LLOV)) [1]. Of the eight known filoviruses, six are known to cause disease in humans. The disease caused by BDBV, EBOV, SUDV, and TAFV is called Ebola virus disease (EVD); that caused by MARV and RAVV is called Marburg virus disease (MVD). However, a majority of filovirus studies have focused on the more famous EBOV, and less attention has been paid to the other pathogenic filoviruses. This review will focus on known immunological, pathogenic, and genomic differences between the filoviruses, and will highlight gaps in knowledge of the non-EBOV filoviruses.

## 2. Diversity among Filoviruses

### 2.1. Prevalence, Endemic Location, and Lethality 

Lethality in human filoviral infections varies considerably (Table 1). Most infections to date have been due to EBOV, which averaged a lethality of ≈79% in the first several outbreaks. However, beginning in late 2013, a massive EVD outbreak in Liberia, Sierra Leone, and Guinea, with limited numbers of cases exported to Nigeria, Mali, Spain, the United Kingdom, Senegal, and the United States, has resulted in lethality approximating 40%. Why this outbreak is less lethal than previous outbreaks is not clear. It is possible that the greater access to health-care facilities and supportive care mitigated the severity of disease, or it could be the larger numbers are more representative of lethality. Genetic variability between outbreaks could also play a significant role in EVD outbreak lethalities, although recent studies indicate that the viruses in the current outbreak are genetically very similar to those found in past EVD outbreaks [2,3,4].

**Table 1:** Location and lethality of filovirus disease outbreaks.

Virus	Genus	Location	% Lethality (Total ^#^ Cases)
**EBOV**	*Ebolavirus*	DRC, Congo, Gabon, Guinea, Sierra Leone, Liberia	42% (29,637) *
**SUDV**	*Ebolavirus*	Sudan, Uganda	54% (792)
**BDBV**	*Ebolavirus*	DRC, Uganda	32% (206)
**TAFV**	*Ebolavirus*	Cote d’Ivoire	0% (1)
**RESTV**	*Ebolavirus*	Philippines, China	0% (11)
**MARV**	*Marburgvirus*	Uganda, Rhodesia, Kenya, DRC, Angola	80% ^§^ (465)
**RAVV**	*Marburgvirus*	Kenya, DRC, Uganda	Unknown ^§^
**LLOV**	*Cuevavirus*	Spain	N/A

* As of 9 September 2015. ^§^ There have been three known instances of RAVV infections in humans; in 1987 (1 patient infected and succumbed), 2007 (1 patient infected and survived), and 1998–2000, when a RAVN and MARV mixed disease outbreak occurred. It is not possible to accurately list the number of infections and lethality of RAVV; the lethality and number of patients for MARV listed here comprises MARV and RAVV disease outbreak statistics combined. There have been no reports of LLOV infection in humans. Lethality data obtained from CDC as reported on www.cdc.gov. ^#^, number; DRC, Democratic Republic of the Congo; N/A, not applicable.

MARV was originally thought to be considerably less lethal than the Ebolaviruses, as the seminal MVD outbreak in the former West Germany and Yugoslavia in 1967 resulted in ≈24% lethality [5,6]. However, these infections were aggressively treated, and in many cases, therapies including infusion of clotting factors or immune sera, which, viewed with current knowledge, could have significantly enhanced the survival rate of these patients. Small and sporadic MVD outbreaks over the next few decades did nothing to alter the perception of lower lethality of MARV. Later, larger outbreaks in the DRC and Angola resulted in similar lethality compared to EBOV [7,8]. Overall, MARV and RAVV infections (≈465) have resulted in ≈80% lethality, virtually indistinguishable from earlier EBOV infections. 

SUDV infections in humans have resulted in 54% lethality in 792 individuals (Table 1). BDBV, a more recently discovered Ebolavirus [9], has infected 206 individuals with a lethality of 32%. There has only been one documented TAFV infection in humans, with that person recovering after a severe illness [10]. However, TAFV has been associated with a precipitous decline in non-human primate (NHP) populations in the wild [10]. Although NHPs readily succumb to infection, there have been no demonstrable illnesses with RESTV infection in humans, despite the development of anti-RESTV antibodies in 11 infected individuals [11]. This has led to widespread predictions that RESTV is not pathogenic in humans. Live LLOV has never been recovered; the virus was discovered by gene sequencing of tissues from bat populations after a massive die-off in Spain [12], and the pathogenicity in humans and NHPs is unknown. 

EVD outbreaks have generally occurred in or around tropical rainforests, while Marburgvirus infections tend to arise in savannah areas, although all filoviruses can be spread by travel of infected individuals. EVD outbreaks have occurred primarily in Equatorial Africa, namely the DRC, Congo, and Gabon [13]. However, a widespread outbreak in 2013–2015 sprouted in Western Africa, including Guinea, Sierra Leone, and Liberia [14]. This recent outbreak has taken place in much more populated cities, including Conakry, the capital of Guinea. SUDV has occurred in Sudan and Uganda [13], while EVD outbreaks due to BDBV infection have been documented in the DRC [15] and Uganda [9]. TAFV is endemic to Côte d’Ivoire [10,16], while RESTV has been found in the Philippines [17] and China [18]. MARV and RAVV infections generally occur in arid woodland African areas [13]. LLOV has only been found in bats in Spain [1]. At this point, it is unknown why the different filoviruses tend to be geographically exclusive. One hypothesis is that the carrier animals, often thought to be bats, may have distinct geographic habitats. It is likely that these generalizations of endemic locations for filovirus will change, as the possible spread of the viruses are dependent on localization of known and purported carriers that may be quite expansive.

### 2.2. Clinical Findings

Early symptoms of filoviral infections include fever, headache, weakness, vomiting, red eyes, and diarrhea [13]. As these early symptoms are relatively non-specific, they are often attributed to other more common endemic infections, such as malaria and typhoid fever. In many cases later stages of infection can consist of petechia along with coagulopathy, thrombocytopenia, and internal and occasionally external hemorrhage. End-stage disease often resembles shock-like symptoms, with hypotension and multiple organ failure. An elevation of aspartate aminotransferase (AST), blood urea nitrogen (BUN), and creatinine (CRE) has been characterized in SUDV infection.

Cerebral edema and encephalopathy have also been noted in filoviral infections [19,20,21,22,23]. Leukopenia, followed by leukocytosis and the presence of activated lymphocytes, often occur in filoviral infections, as do increases in AST and alanine aminotransferase (ALT). In a single RAVV case that was clinically observed, there were similar symptoms as those found in MARV infections [24]. During the current EVD outbreak, live virus was detected months after clinical resolution in the intraocular aqueous humor of an individual [25]. EBOV in semen has also been detected by PCR or, in some cases, live virus isolation after disease remission [26], and live SUDV has been isolated from breast milk [27] of a convalescent individual. MARV has been isolated from the semen [28] and ocular fluid [29] of convalescent patients. These reports demonstrate the need for continued surveillance of recovering individuals and their contacts.

Overall, there are no clearly-defined differences in clinical disease course between different pathogenic filoviral infections in humans. This is likely to change with additional published literature in current and future filovirus human infections. Indeed, future research should attempt to discover whether there are significant differences between clinical course and pathogenesis during different filovirus infections in humans.

### 2.3. Cytokine Responses and Human Genetics

Studies from human outbreaks have suggested a connection between cytokine production and lethality (Table 2). In EBOV infection, pro-inflammatory cytokine production is correlated with lethality, as TNF-α, IFN-γ, IL-1β, IL-6, IL-8, MCP1, MIP-1α, and MIP-1β levels are increased in fatal versus non-fatal infection [30,31,32,33]. An early induction in pro-inflammatory cytokine and chemokine levels is also evident in asymptomatic infected individuals; however, this induction is transient as these levels return to baseline soon after infection [34,35]. In addition to pro-inflammatory cytokines, the production of the anti-inflammatory mediators IL-10 and nitric oxide are observed in fatal infections, possibly as a negative feedback response to attempt to dampen the high pro-inflammatory environment [31,33]. Similarly, in SUDV infection, the pro-inflammatory cytokines IL-1α, IL-6, MCP-1, M-CSF, MIP-1α, and IL-8 are increased in fatal infection, as is nitric oxide [36,37]. However, TNF-α and IFN-γ, while elevated in fatal EBOV infections, are not known to be higher in fatal SUDV infection. SUDV survivors do have elevated sCD40L and IFN-α expression compared to non-survivors. Data from human BDBV infections indicated that fatalities were associated with increased IFN-γ and IL-10 levels; however, high levels of IL-1α, IL-1β, and IL-6 were not found in either fatal or non-fatal cases [38]. Together, these data roughly correlate with the theory that uncontrolled pro-inflammatory cytokine production and low type I IFN responses contribute to filovirus lethality [39,40]. Indeed, MARV infection of guinea pigs was shown to be partially controlled by neutralization of TNF-α [41].

**Table 2:** Cytokine responses and human genes associated with lethal *vs.* non-lethal filovirus infections in humans.

Virus	Lethal	Non-Lethal	References
**EBOV**	TNF-α, IFN-γ, IL-1β, IL-6, IL-8, MCP1, MIP-1α, MIP-1β, IL-10		[30,31,32,33]
**SUDV**	IL-1α, IL-6, MCP-1, M-CSF, MIP-1α, Thrombomodulin, ferritin, D-Dimer, AST, BUN, CRE, IL-10, IL-8, NO, HLA-B*67, HLA-B*15	sCD40L, CA++, IFN-α, HLA-B*07, HLA-B*14	[36,37]
**BDBV**	IFN-α2, IFN-γ, IL-10		[38]
**TAFV**	ND	ND	
**RESTV**	ND	ND	
**MARV**	ND	ND	
**RAVV**	ND	ND	
**LLOV**	N/A	N/A	

N/A, not applicable; ND, not determined.

There are few published data on cytokine levels in human MARV, RAVV, TAFV, or RESTV cases, although the study of a single MARV fatal infection showed a similar pro-inflammatory cytokine profile to that of EBOV during infection [22]. Animal model studies of MARV, RAVV, and RESTV also exhibit increased pro-inflammatory cytokine production in lethal infections [42,43,44,45,46,47,48,49]. However, these studies must be viewed in a different light, as these animal models are almost completely universally lethal (with the noted exception of TAFV and BDBV), making it difficult to compare lethal to non-lethal infection. However, infection of mice with mouse-adapted RAVV induces increased IFN-γ, IL-5, IL-12, CCL2, and CXCL9 relative to infection with non-lethal RAVV [49]. Similarly, mouse-adapted MARV infection leads to elevated levels of multiple pro-inflammatory cytokines (and elevated IL-10), compared to non-lethal wild-type MARV infection of mice [50].

CD8 T cells and NK cells are thought to contribute to protection during filovirus infection. MHC I alleles have been correlated with an increased chance of death or survival after SUDV infection; HLA-B*07 and HLA-B*14 are strong predictors for survival, while HLA-B*15 and HLA-B*67 correlate with lethal infections [51]. In a cohort with approximately 55% survival, all 11 patients with HLA-B*07 survived, and interestingly, epitope prediction software identified several EBOV epitopes predicted to bind to HLA-B*07, although no further analyses were undertaken. As no genetics studies have been performed with other filoviruses, future studies should seek to examine the possible role of genetics in effective or deficient immune responses to filovirus infection. 

### 2.4. Type I IFN Antagonism and Requirements

Type I IFN is a primary defense of the innate immune system against viral infections. The production of Type I IFN during infection has broad effects on global transcription, activation of the innate and adaptive immune responses, and induction of multiple direct anti-viral cellular pathways. Type I IFN appears to be vital in controlling filovirus infections. This was originally discovered in mouse models, where wild-type filoviruses are not pathogenic; however, mice lacking type I IFN signaling were found to be largely susceptible to wild-type filovirus infections [52,53,54,55]. Similarly, in a seminal reverse genetics study, the pathogenesis of mouse-adapted EBOV was found to be strongly correlated with functional resistance to type I IFN in mouse cells [56]. There is also evidence that RESTV infection *in vitro* is less able to inhibit type I IFN responses compared to EBOV or MARV, suggesting a possible mechanism for its diminished pathogenicity in humans [57].

Filoviruses can inhibit type I IFN production and signaling in a number of ways (Table 3). EBOV VP24 blocks type I IFN signaling via inhibition of STAT1 translocation to the nucleus [58,59], while EBOV VP35 blocks induction of type I IFN responses through inhibition of IRF-3 and IRF-7 activation [60,61,62]. MARV, however, inhibits type I IFN signaling by blocking of STAT1, STAT2, STAT3, Jak1, and Tyk2 signaling through VP40 [63], and via coating of viral dsRNA through VP35 [64]. RAVV VP40 has also been shown to block type I IFN signaling by inhibiting Jak1 activation [65].

**Table 3:** Type I IFN inhibition by filoviruses.

Virus	VP35	VP40	VP24
**EBOV**	Type I IFN antagonism		Type I and II IFN antagonism
**SUDV**	ND	ND	ND
**BDBV**	ND	ND	ND
**TAFV**	ND	ND	ND
**RESTV**	Type I IFN antagonism	ND	Type I and II IFN antagonism
**MARV**	Type I IFN antagonism	Type I/II IFN antagonism	
**RAVV**	ND	Type I/II IFN antagonism	
**LLOV**	ND	ND	ND

ND, not determined.

Treatment of NHPs with type I IFN as a monotherapy has shown moderate efficacy against MARV, with an increase in time-to-death [66]. Similarly, in EBOV infection only an increase in time-to-death but no increase in survival has been described after type I IFN therapy [67]. However, type I IFN as an adjuvant therapy in combination with antibody therapy has shown greater protection in preliminary NHP studies [68]. Since filoviruses can block both the induction and signaling of type I IFN, utilizing exogenous type I IFNs as a monotherapy may not be sufficient to overcome the inhibition.

It is important to note that viral inhibition of type I IFN is well characterized in EBOV, MARV, and RAVV, with very limited data for RESTV. Potential differences in type I IFN inhibition by SUDV, BDBV, LLOV and TAFV are an understudied area of filovirus biology that should be actively pursued in future studies.

### 2.5. Animal Models

The use of animal models has been indispensable in understanding basic pathogenesis of filoviral disease, and in the testing and fine-tuning of vaccines and therapeutics (Table 4). All Marburg- and Ebolaviruses are pathogenic in non-human primates, although with varying degrees of lethality, regardless of the route of administration. EBOV, SUDV, RESTV, MARV, and RAVV are generally 100% lethal after infection of non-human primates in the laboratory, whereas TAFV and BDBV appear to be less pathogenic (with 67%–75% lethality in limited studies [69,70]). However, early studies often describe NHP mortality lower than 100% with filovirus infection, which could possibly be attributed to less strict euthanasia criteria in past studies. While non-human primates are indispensable for filovirus challenge studies, there are differences between pathogenesis in primates of various species, and their high expense requires additional animal models for experimentation.

**Table 4:** Animal models for filovirus infection.

Virus	NHP Models (WT Virus)	Guinea Pig Models (Guinea Pig-Adapted Virus)	Hamster Models	Mouse Models (Mouse-Adapted Virus)	Mouse Models (WT Virus)
**EBOV**	Rhesus macaque [71,72], cynomolgous macaque [73], grivets [73,74], baboon [75,76]	Yes [77,78]	Yes [79]	Yes [80]	Yes IFNAR [52], STAT1 [53]
**SUDV**	Rhesus macaque [81], cynomolgous macaque [73]	No	No	No	Yes IFNAR [52], STAT1 [53]
**BDBV**	cynomolgous macaque [70]	No	No	No	No
**TAFV**	cynomolgous macaque [69]	No	No	No	No
**RESTV**	Rhesus macaque [82], cynomolgous macaque [73,83], grivets [73]	No	No	No	Yes STAT1 [53]
**MARV**	Rhesus macaque [84], cynomolgous macaque [85], grivets [84], marmosets [74]	Yes [6,86]	No	Yes [87]	Yes IFNAR [52], STAT1 [53]
**RAVV**	Rhesus macaque [24], cynomolgous macaque [88]	Yes [86]	No	Yes [49]	Yes IFNAR [52], STAT1 [53]
**LLOV**	No	No	No	No	No

Natural RESTV infections have been discovered in domestic swine in China and the Philippines [18,89]. Interestingly, these infections were only found in swine that were co-infected with porcine reproductive and respiratory syndrome virus (PRRSV), which alone causes severe respiratory disease. Laboratory RESTV infection of young pigs resulted in viral replication but no overt symptoms [90]. Experimental EBOV infection of young pigs, however, resulted in severe lung pathology and viral transmission [91,92,93]. Additional experiments studying the pathogenicity of RESTV in pigs of different species, as well as testing SUDV, BDBV, TAFV, MARV, and RAVV for pathogenesis in pigs, would clarify the role of these animals as possible filovirus reservoirs.

Although wild-type MARV, RAVV, EBOV, SUDV, and RESTV do replicate in mouse models, these viruses are not lethal in adult wild-type mice [52,53,54,55]. In guinea pigs, wild-type EBOV, MARV, and RAVV cause a transient febrile illness, and several passages through these animals resulted in lethal guinea pig-adapted viruses [6,78,86]. There are no reports of TAFV or BDBV replication or pathogenesis in any rodent models. The development of a mouse-adapted EBOV [80] through serial passaging in progressively older mice allowed for exploitation of the vast resources available in mice, including genetic knockouts and immune detection reagents. Additionally, different strains of mice have varying susceptibilities to infection with certain strains more closely mimicking human infections [94]. Furthermore, mouse-adapted EBOV is useful as a lethal virus in a novel hamster model which has many similarities to human infection [79]. A more recent set of papers describe the generation of MARV and RAVV adapted to be lethal in mice [49,50,87]. However, there are no rodent-adapted viruses for SUDV, BDBV, TAFV, RESTV, or obviously LLOV (since no live LLOV has yet been isolated). Nonetheless, mice lacking type I IFN responses are susceptible to wild-type EBOV, SUDV, RESTV, MARV, and RAVV viruses [52,53,54,55]. Therefore, additional small animal models are needed, especially for SUDV, BDBV, and TAFV infections. As successful models of EBOV and MARV utilized serial passaging in wild-type or SCID mice, these methods could be used to develop additional mouse-adapted filoviruses. 

Rodents are valuable for the development and characterization of vaccines and therapeutics, which can then be further validated in the more expensive and stringent NHP models [95]. Although rodent models are similar in viral tropism and induction of immune responses, NHP models most closely mimic human infections and are vital to the development of effective vaccines and therapeutics against filovirus infections.

Therefore, while EBOV, RAVV and MARV are well-represented in multiple animal models, there are fewer models available for RESTV, SUDV, TAFV, and BDBV. There are no models for LLOV, due to the lack of a characterized live virus.

### 2.6. Gene Organization

Although the overall genomic structure of filoviruses is generally conserved, there are some differences in the number of gene overlaps, number of transcripts, and glycoprotein (GP) transcripts (Table 5). GP is responsible for attachment and entry of the virions into host cells. Attachment of EBOV to cells has been purported to occur via multiple cell surface proteins, including C-type lectins, β1 integrins, TAM receptor protein tyrosine kinases, and TIM-1 protein. Nonetheless, a clear cell surface receptor for filoviruses has not been elucidated [13]. However, entry of EBOV, SUDV, BDBV, TAFV, MARV, and LLOV into the cytoplasm from the endosome appears to be dependent on the presence of Niemann-Pick C1 (NPC1) [96,97,98]. 

There are major differences in the GP forms between filoviruses (Table 5). While all filoviruses generate the transmembrane GP (made from a trimer of GP1,2 dimers; in EBOV, but not MARV or RAVV, this form is made after transcriptional editing of GP), EBOV also produces additional forms of GP. Soluble GP (sGP) is released from the infected cells and circulates in the bloodstream. A cleavage product from sGP called δ-peptide is also made [13,99]. EBOV also generates a small soluble GP (ssGP) via transcriptional editing, which is expressed as a homodimer [13,99,100]. There is some evidence that sGP and ssGP have differing biological effects [101]. The functions of sGP and ssGP are not well-known, although it has been suggested that they can act as decoy targets for neutralizing antibodies [102], activators of excessive inflammation [103], and induce effects on endothelial cell permeablization during infection [104]. SUDV, TAFV, BDBV, LLOV, and RESTV are predicted to generate sGP, ssGP, and δ-peptide based on sequence similarity, but their discovery has not been published. Strikingly, however, Marburgviruses lack sGP, ssGP, and Δ-peptide, yet retain similar or enhanced pathogenicity compared to Ebolaviruses [13]. Studies analyzing in vivo pathogenicity of guinea pig-adapted EBOV lacking production of sGP have yielded conflicting results [105,106]. The in vivo relevance of sGP and ssGP of Ebolaviruses is therefore not yet fully understood. Since generation of viruses that lack the ability to make sGP has been accomplished for EBOV [107], infection in animal models with additional modified Ebolaviruses is important to determine the role of these factors during infection.

**Table 5:** Difference in genetic structure amongst filoviruses.

Virus	^#^ of Gene Overlaps	^#^ of Transcripts	GP Gene Products	Reverse Genetics Systems
**EBOV**	2 or 3	7	GP, sGP, ssGP, Δ-peptide	Full-length clone; minigenome
**SUDV**	3	7	GP, sGP, ssGP, Δ-peptide (predicted)	
**BDBV**	3	7	GP, sGP, ssGP, Δ-peptide (predicted)	
**TAFV**	3	7	GP, sGP, ssGP, Δ-peptide (predicted)	
**RESTV**	2	7	GP, sGP, ssGP, Δ-peptide (predicted)	Minigenome
**MARV**	1	7	GP	Full-length clone; minigenome
**RAVV**	1	7	GP	
**LLOV**	4	6 (predicted; VP24 and L are from same dicistronic transcript)	GP, sGP, ssGP, Δ-peptide (predicted)	

#, number.

### 2.7. Vaccines

A number of vaccines have been shown to be effective in NHP or rodent filovirus models, and this is reviewed in more detail elsewhere [108,109]. A variety of platforms have been tested, including replicating pseudotyped vectors (vesicular stomatitis Indiana virus (VSV), human parainfluenza virus 3 (HPIV-3)), proteinaceous vaccines (virus-like particles (VLP), GP protein), DNA-based vectors, single-cycle pseudotyped vectors (Venezuelan equine encephalitis virus replicons (VRP), Kunjin replicon VLPs [110], adenovirus (AdV), and inactivated virus preparations (Table 6). These vaccines are mostly based on the generation of immune responses to GP. Most of the work done in the vaccine area has focused on EBOV, followed by MARV and RAVV. Some work has been done for SUDV and BDBV, while very little has been published on TAFV. No vaccines have been studied for protection against RESTV or LLOV, although it has been shown that there is no cross-reactivity of anti-LLOV antibodies against other filoviruses [99]. VSV vectors have been protective in NHPs when given post-infection (MARV and EBOV), and AdV and VLPs have been shown to protect mice when given after infection. Both the VSV and AdV platforms are currently being tested in human clinical trials against EBOV [111,112,113,114,115].

### 2.8. Therapeutics

Several therapeutics have been shown to be effective in animal models of filovirus infections; again, most of the studies have focused on EBOV and MARV.

#### 2.8.1. Antibodies

The history of antibody-based treatment of filovirus infections began with the original MVD outbreak in Germany and the former Yugoslavia in 1967. Four MARV patients received convalescent sera from survivors and had a mild disease course, although controlled experiments were understandably not performed [21]. Later, several rodent studies showed that antibodies could be protective against EBOV and MARV. In the mid-1990s investigators found that passive transfer of hyperimmune horse sera was protective against EBOV pathogenesis in baboons [116,117]; however, similar sera preparations were not protective against EBOV in cynomolgous macaques, although viral titers were dramatically decreased during the first several days of infection [67]. A neutralizing antibody (KZ52) from a human survivor protected against EBOV in rodent models but was reported to be not protective in rhesus macaques [118], further dampening enthusiasm for antibody therapy of filovirus infections. 

A seminal study in 2012 showed that passive transfer of IgG from immunized cynomolgous macaques could protect naïve cynomolgous macaques from EBOV or MARV infection, even when given 48 hours after infection [119]. Following this, a number of studies in macaques found partial to complete protection against EBOV with monoclonal antibody cocktails (consisting of two or three different antibodies) [120,121,122], though not as effective at 48 hours post-infection when compared to polyclonal antibodies [119]. Optimized cocktails have now been demonstrated to have remarkable efficacy as far as five days after Infection [4]. These cocktails, and plasma from convalescent patients, have been used sparingly in humans during the current EVD outbreak [123,124]. Despite these advances, there are no new human clinical data on the efficacy of these antibody therapies. Indeed, much has yet to be determined regarding the use of these therapies in human infections. As long lasting antibody responses have been observed in past filovirus survivors, researchers will continue to evaluate the humoral immunity in the survivors of the current outbreak.

In addition, no studies have been published on protective monoclonal antibody therapy in NHPs for MARV, RAVV, SUDV, BDBV, TAFV, or RESTV infections; also lacking are polyclonal antibody therapies for RAVV, SUDV, BDBV, TAFV, or RESTV. Following the striking successes of antibody-mediated therapies in animal models of EBOV infections, future work should be aimed at the generation of protective antibody responses to these other filoviruses.

**Table 6:** Effective vaccines and therapies in animal models of filovirus infection.

Virus	Vaccines	Antibody Therapy	Antisense Therapy	Small Molecules
**EBOV**	VLP, VRP, DNA, AdV, VSV, HPIV, protein, DNA/AdV, Kunjin replicon, VRP	Polyclonal, monoclonal	PMO, siRNA	FG-103, BCX4430, NSC 62914, FGI-106
**SUDV**	VSV, AdV, VRP			
**BDBV**	VSV, DNA/AdV			
**TAFV**	VSV			
**RESTV**				
**MARV**	VSV, VLP, DNA, killed, protein	Polyclonal	PMO, siRNA	BCX4430
**RAVV**	VSV, VLP, DNA, killed, protein		PMO, siRNA	FG-103, BCX4430, NSC 62914
**LLOV**				

PMO, Phosphorodiamidate Morpholino Oligomers.

#### 2.8.2. Small Molecules

A recent study has shown remarkable protection in macaques with the synthetic adenosine analog, BCX4430. Macaques infected with MARV were completely protected when treatment began 48 hours after infection [125]. BCX4430 also protected mice from RAVV and guinea pigs from MARV infection when given up to 72 hours after infection; mice were also protected from EBOV infection [125]. Other small molecules that protect in mouse or guinea pig models have been shown for EBOV and RAVV. An antioxidant molecule NSC 62914 protected mice from EBOV or RAVV infection [126]. Although the mechanism of action is currently unknown, FG-103 protected mice from EBOV or RAVV infection when given 24 hours after infection [127]. FG-106 also protected mice from EBOV given 24 hours after infection [128]. Another adenosine analog, 3-deazaneplanocin A, was shown to protect mice from EBOV [129] by induction of type I IFN [130].

#### 2.8.3. Antisense

The use of antisense RNA therapies has recently gained increased attention as a highly specific potential therapy for filovirus infections. The use of short interfering RNA (siRNA) encapsulated in lipid formulations has been shown to be effective against MARV infection in non-human primates when given post-exposure [131], and against MARV or RAVV in guinea pigs [132]. Lipid-encapsulated siRNA was also protective against EBOV in non-human primates [133].

Phosphorodiamidate Morpholino Oligomers (PMO) are synthetic, stable, single-stranded RNA-like molecules that function as antisense therapies, without the need for lipid-based delivery. PMOs against one or two gene targets have been shown to protect non-human primates from EBOV and MARV infection [134,135,136,137] and against RAVV in mice [134]. PMOs have also been developed to target host antigens; unlike direct viral targeting, this approach would not put selective mutational pressure on the virus. A PMO targeting HSPA5 has shown strong efficacy in a mouse model of EBOV infection [138]. Despite the numerous successful antisense therapies for EBOV, MARV and RAVN, to date, no antisense studies have been published for SUDV, TAFV, BDBV, or RESTV.

#### 2.8.4. Clotting Factors

In the original 1967 MVD outbreaks, many patients received treatments to alleviate the bleeding problems observed, including fibrinogen, clotting factors, vitamin K, and blood transfusions. Whether this was effective in ameliorating the viral pathogenesis is not known, although the overall lethality of this outbreak was much lower than in later outbreaks where patients did not receive this level of care. Interestingly, experiments in non-human primates focused on infusion of anti-coagulants, including rNAPC2 and APC, provided only limited protection against EBOV or MARV ranging from 17%–33% [139,140,141].

Additional work analyzing the effect of transfer of pro-clotting factors, as was done in 1967 for MARV, would be informative for future treatment guidelines for filovirus infections.

### 2.9. Research Tools Available

#### 2.9.1. Purified Proteins, Antibodies and ELISA kits.

Given the enhanced interest in filovirus research, access to reagents for these viruses is imperative. There are numerous antibodies available to Ebola-, Marburg-, and cuevaviruses. There is little cross-reactivity of known antibodies between these three viruses. A large number of reagents exist for EBOV, MARV and SUDV, while there are considerably fewer available reagents for the other filoviruses. There are no commercially available monoclonal antibodies for BDBV or LLOV and no recombinant viral proteins or viral protein specific monoclonal antibodies for RAVV, TAFV, LLOV, or RESTV. As strong mechanistic studies heavily rely on these basic reagents an effort should be made to widen the availability of reagents to these additional filoviruses. In response to the current EVD outbreak, the NIH has established the Viral Hemorrhagic Fever Immunotherapeutic Consortium which has the potential to develop into a valuable asset in the development and characterization of filoviral-specific antibodies. The commercially available reagents are listed in Table 7.

**Table 7:** Reagents commercially available for filovirus studies. * = GP1,2 without transmembrane domain. N/A, not available. Updated as of 9 September 2015.

Virus	Recombinant Protein (Modification)	Polyclonal Antibodies	Monoclonal Antibodies	ELISA Circulating Antibodies (Animal)	ELISA Circulating Antigen
**EBOV**	GP*, GP*(His),VP40, GP1, GP2 , GP, NP, VP24, VP40	Rabbit αVP40, αNP, αGP, αGP(Biotin), αVP35, αL	Mouse αGP, Chimeric αGP IgG (c6D8, c6D8, h13F6, h13C6-FR1, 7C2G10, 6F9D2, 1F6E9, 7B2B5, 2A5D12, 7B5D1, 3C4G3, 4F3), Human (KZ52)	NP (mouse), GP (human), VP40 (mouse)	GP
**SUDV**	GP*(His), GP1, GP2	Rabbit αVP40, αNP, αGP	αVP40 (1G10), αGP* (2H5, 6D11, 15H10)	GP (mouse, human and NHP)	N/A
**BDBV**	GP*(His), GP1, GP2	Rabbit αGP	N/A	N/A	N/A
**TAFV**	N/A	N/A	N/A	N/A	N/A
**RESTV**	GP*(His)	Rabbit αGP	N/A	N/A	N/A
**MARV**	GP*(His) Angola, GP*(HA) Musoke	Rabbit αVP40, αNP, αGP, αGP(Biotin),	αVP40 (1D1, 6B1), sGP*(5C1)	GP (mouse, human and NHP)	N/A
**RAVV**	N/A	N/A	N/A	N/A	N/A
**LLOV**	N/A	N/A	N/A	N/A	N/A

N/A, not applicable.

#### 2.9.2. Reverse Genetics Systems

Reverse genetics systems have been established for EBOV [142] and MARV [143], but are lacking for the other filoviruses (Table 5). Minigenome systems have been established for EBOV, MARV, and RESTV only.

#### 2.9.3. Diagnostics

Since filovirus infections present with non-specific symptoms similar to many other infections, specific tests are required for accurate diagnostics. This can be achieved through the use of reverse-transcription polymerase chain reaction (RT-PCR) from patient samples, or detection of filovirus antigens via the use of antigen capture ELISA. Post-hoc surveillance for filovirus infection in survivors can be assessed by measurement of antibodies against filovirus proteins through ELISAs or western blotting. Finally, functional virus can be detected by plaque assays or NHP infection using BSL-4 facilities. Molecular diagnostics mentioned above are available for all filoviruses.

## 3. Conclusion

Impressive progress has been made in understanding the basic virology, pathogenesis, and vaccine and therapeutic development for EBOV, and, to a lesser extent, MARV and RAVV. RESTV is not known to be pathogenic in humans; this likely has dampened research into this filovirus. Whether the newly discovered LLOV is pathogenic in higher animals and primates will also determine how well-studied this virus will be. As highlighted throughout this article, many gaps in our knowledge of SUDV, BDBV, and TAFV persist. As past, current, and future studies largely focus on EBOV, it will be important to expand our knowledge of the other filoviruses in order to be prepared in the case of large future outbreaks.
